# Erratum to Stretococcus *gallolyticus* infective endocarditis, a different presentation-a case report

**DOI:** 10.1097/CP9.0000000000000045

**Published:** 2023-04-04

**Authors:** Cardiology Plus Editorial Office

In the article entitled “Stretococcus *gallolyticus* infective endocarditis, a different presentation-a case report”^[1]^ published in *Cardiology Plus* 7(3):p 144-147, July-September 2022, it stated “We present a case (57-year-old man) of infective endocarditis caused by Streptococcus gallolyticus in a patient with incident early-stage colon cancer.” in the abstract section, the “57” should be corrected to “68.” The publisher sincerely regrets the error.
